# BRD4 Inhibitor Inhibits Colorectal Cancer Growth and Metastasis

**DOI:** 10.3390/ijms16011928

**Published:** 2015-01-16

**Authors:** Yuan Hu, Jieqiong Zhou, Fei Ye, Huabao Xiong, Liang Peng, Zihan Zheng, Feihong Xu, Miao Cui, Chengguo Wei, Xinying Wang, Zhongqiu Wang, Hongfa Zhu, Peng Lee, Mingming Zhou, Bo Jiang, David Y. Zhang

**Affiliations:** 1 Guangdong Provincial Key Laboratory of Gastroenterology, Department of Gastroenterology, Nanfang Hospital, Southern Medical University, 1838 North Guangzhou Road, Guangzhou 510515, China; E-Mails: drhuyuan@gmail.com (Y.H.); zhoujieqiong2008@sina.com (J.Z.); sunwingwxy@163.com (X.W.); wangzq815@gmail.com (Z.W.); 2 Department of Pathology, Icahn School of Medicine at Mount Sinai, New York, NY 10029, USA; E-Mails: fei.ye@mssm.edu (F.Y.); miao.cui@mssm.edu (M.C.); hongfa.zhu@mountsinai.org (H.Z.); 3 Department of Medicine, Immunology Institute, Icahn School of Medicine at Mount Sinai, New York, NY 10029, USA; E-Mails: huabao.xiong@mssm.edu (H.X.); liang.peng@mssm.edu (L.P.); feihong.xu@mssm.edu (F.X.); 4 College of Arts and Sciences, University of North Carolina at Chapel Hill, Chapel Hill, NC 27514, USA; E-Mail: zihanz@live.unc.edu; 5 Departments of Medicine Bioinformatics Core, Icahn School of Medicine at Mount Sinai, New York, NY 10029, USA; E-Mail: chengguo.wei@mssm.edu; 6 Departments of Pathology, Urology, NYU Cancer Institute, New York Harbor Healthcare System, New York University, School of Medicine, New York, NY 10010, USA; E-Mail: Peng.Lee@nyumc.org; 7 Department of Structural and Chemical Biology, Icahn School of Medicine at Mount Sinai, New York, NY 10029, USA; E-Mail: ming-ming.zhou@mssm.edu

**Keywords:** colorectal cancer, BRD4, MS417, metastasis, epithelial-to-mesenchymal transition (EMT)

## Abstract

Post-translational modifications have been identified to be of great importance in cancers and lysine acetylation, which can attract the multifunctional transcription factor BRD4, has been identified as a potential therapeutic target. In this paper, we identify that BRD4 has an important role in colorectal cancer; and that its inhibition substantially wipes out tumor cells. Treatment with inhibitor MS417 potently affects cancer cells, although such effects were not always outright necrosis or apoptosis. We report that BRD4 inhibition also limits distal metastasis by regulating several key proteins in the progression of epithelial-to-mesenchymal transition (EMT). This effect of BRD4 inhibitor is demonstrated via liver metastasis in animal model as well as migration and invasion experiments* in vitro*. Together, our results demonstrate a new application of BRD4 inhibitor that may be of clinical use by virtue of its ability to limit metastasis while also being tumorcidal.

## 1. Introduction

Colorectal cancer (CRC) is the third most prevalent form of cancer globally and accounts for approximately 8% of all estimated new cases of cancer in 2014 [1]. Currently, CRC is commonly treated using combinations of surgery, chemotherapy and radiotherapy. However, chemotherapy drugs, such as fluorouracil (5-FU) and irinotecan (commonly used as part of the FOLFIRI regimen), have negative effects on healthy tissue at the doses required for successful tumor elimination and may also be promoting pathways that lead to tumor resistance, through the activation of pro-inflammatory and anti-apoptotic factors [2,3,4]. Surgical interventions may not be able to clear out all cancerous tissue, and the mortality rate of CRC, especially in cases with distant metastases, still remains high [1]. As a result, it is clear that novel therapies for treating CRC that target the tumor cells in different ways are urgently needed.

Epigenetic modifications, such as DNA methylation and posttranslational histone modifications, have been shown to affect almost every component of gene regulation. Histone (de)acetylation, which functions importantly in chromatin remodeling and gene activation [5], is best characterized in CRC [6]. In 1999, bromodomains (BRDs), a group of protein modules which contain ~110 amino acids were reported as the first protein modules to recognize acetylated lysine in histone through the unusual left-handed four-helix bundle structure [7,8]. To date, a total of 61 BRDs have been clustered into eight human BRD families based on structure and sequence similarity [9]. The bromodomain and extra terminal (BET) family including BRD2, BRD3, BRD4 and BRDT is one of them and features a distinctive domain architecture of two tandem amino-terminal bromodomains that exhibit high levels of sequence conservation [10]. BRD2 and BRD4 have been reported to be involved in cell cycle progression, given the evidence that binding of BRD2 and BRD4 to acetylated chromatin persists even during mitosis when chromatin is highly condensed and transcription is interrupted [11,12]. BRD2 is underexpressed in some subtypes of human lymphoma [12]. Recently, BRD4 has been demonstrated to play a key role in the pathogenesis of several different types of cancers and is considered to be a compelling therapeutic target [10]. BRD4 has been identified functioning in an aggressive form of human squamous carcinoma as a component of a recurrent t(15;19) chromosomal translocation [13,14]. It has been found to be involved in a wide range of immune-cell related cancers including acute myeloid leukemia (AML), mixed lineage leukemia (MLL) and diffuse large B cell lymphoma [15,16,17,18]. Recently, BRD4 has also been of great interest in diverse solid tumors. Many of those, such as non-small cell lung cancer, metastatic breast cancer and melanoma do not currently have effective conventional therapies, making BRD4 inhibition a very promising option by default [19,20,21,22,23].

The naturally existing isoforms of BRD4 and their functions are not fully understood since Scott R. Floyd and his groups [24] have shown that BRD4 encodes A, B and C three splice isoforms with two bromodomains and an extra-terminal (ET) domain presenting in each isoform. They demonstrated that the A isoform contains a carboxy-terminal domain (CTD), which is notably absent in the B and C isoforms, and it is replaced with a divergent short 75 amino-acid segment in the B isoform. Moreover, only the B isoform has the ability to inhibit DNA damage response signaling by recruiting the condensing II chromatin remodeling complex to acetylated histones through BRD interactions, leading to an uncontrolled cell-cycle progression. While other groups provided the evidences that the two isoforms of BRD4 (short isoform and long isoform) share the same *N*-terminal region except for the final three amino acids and have opposing roles in breast cancer growth and progression [20,21,22].

Motivated by the above rationale, small molecular selective inhibitors of bromodomains in BET proteins have been developed and it is likely that they will find broad application in medicine and basic research as exemplified by the recent significant effects in treating several disorders. Suppression of BRD4 using small compounds such as JQ1, I-BET151 and MS417 has been shown profound efficacy against a wide range of cancers such as diffuse large B cell lymphoma, AML, MLL, non-small cell lung cancer, breast cancer, pancreatic cancer and melanoma [10,15,16,19,23,25]. However, the role of BRD4 has not been well studied for colorectal cancer, with the exception of one paper demonstrated that overexpression of BRD4 reduced colorectal tumor growth* in vivo* [26]. In this paper, we demonstrate that BRD4 inhibition has a significant effect on CRC, and that it can curtail associated tumor metastasis.

## 2. Results and Discussion

### 2.1. BRD4 Is Highly Expressed in Colon Cancer Cells and Colon Cancer Tissues

**Figure 1:**
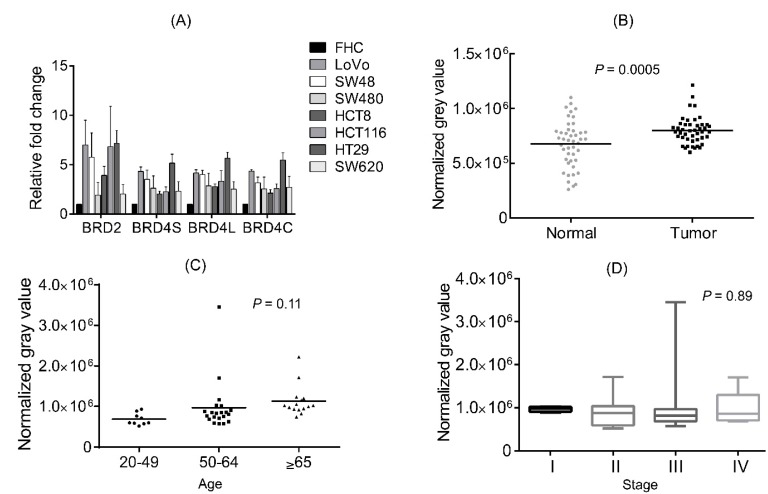
BRD4 is highly expressed in colorectal cancer (CRC). (**A**) mRNA expression levels of BRD2, BRD4 short isoform and BRD4 long isoform in normal colon epithelial cell lines and colon cancer cell lines; (**B**) Protein levels of BRD4 in normal and colon cancer human tissues (tumor and healthy control); (**C**) Protein levels of BRD4 according to different ages; (**D**) Protein levels of BRD4 according to the stage of CRC (per AJCC guidelines). BRD4S: BRD4 short isoform; BRD4L: BRD4 long isoform; BRD4C: Commercial BRD4 primer as positive control; CRC: colorectal cancer.

Since BRD4 has been implicated to be a critical player in the aforementioned cancers, we first explored to see if its expression was also of significance in CRC. Seven established colon cancer cell lines (LoVo, SW48, SW480, HCT8, HCT116, HT29 and SW620) were analyzed for expression of BRD4 and BRD2 relative to the normal colon cell FHC line by real-time PCR. Our results indicate that high levels of BRD2, as well as BRD4 isoforms (long and short) were present in colon cancer cells, as compared to normal colon epithelial cell (Figure 1A). To further confirm this finding, we analyzed expression of BRD4 on the protein level in colon cancer tissues. 45 paired samples of cancerous and healthy colon tissue from patients of different age, gender, disease state, and disease site were analyzed for BRD4 expression by Western blotting (Table 1). Overall, there was a noticeable higher expression of BRD4 in the tumor samples compared to healthy control (*n* = 45; *p* = 0.0005) (Figure 1B). Our analysis also revealed a potential age correlation for BRD4 expression, with older patients tending to have higher expression of the protein (*n* = 45, *p* = 0.11) (Figure 1C). More samples would be needed for confirmation. However, it seemed to be no correlation between the expression level of BRD4 and CRC stages (*n* = 45, *p* = 0.89) (Figure 1D).

**Table 1:** Patients’ clinicopathological characteristics and Brd4 expression fold changes.

Clinicopathological Characteristics		Number (%) of Patients (*n* = 45, Paired)
Age (years)	20–49	9 (20.0)
	50–64	20 (44.4)
	≥65	16 (35.6)
Gender	Male	27 (60.0)
	Female	18 (40.0)
Site of tumor	Ascending colon	6 (13.3)
	Transverse colon	3 (6.7)
	Descending colon	3 (6.7)
	Sigmoid colon	13 (28.9)
	Rectum	20 (44.4)
Histologic differentiation	Well and moderately differentiated (≥50% gland formation)	37 (82.2)
	Poorly differentiated (<50% gland formation)	7 (15.6)
	Missing	1 (2.2)
AJCC stage	I	4 (8.9)
	II-A	6 (13.3)
	II-B	11 (24.4)
	III-A	0 (0.0)
	III-B	13 (28.9)
	III-C	5 (11.1)
	IV	6 (13.3)
T classification	T1	1 (2.2)
	T2	3 (6.7)
	T3	12 (26.7)
	T4	29 (64.4)
N classification	N0	21 (46.7)
	N1	15 (33.3)
	N2	9 (20.0)
M classification	M0	39 (86.7)
	M1	6 (13.3)
Relative fold change of cases expressing Brd4 (Tumor/Normal)	>1	31 (68.9)
	<1	14 (31.1)

### 2.2. Colon Cancer Cell Proliferation Is Potently Inhibited by BRD4 Inhibitor in Vitro

**Figure 2:**
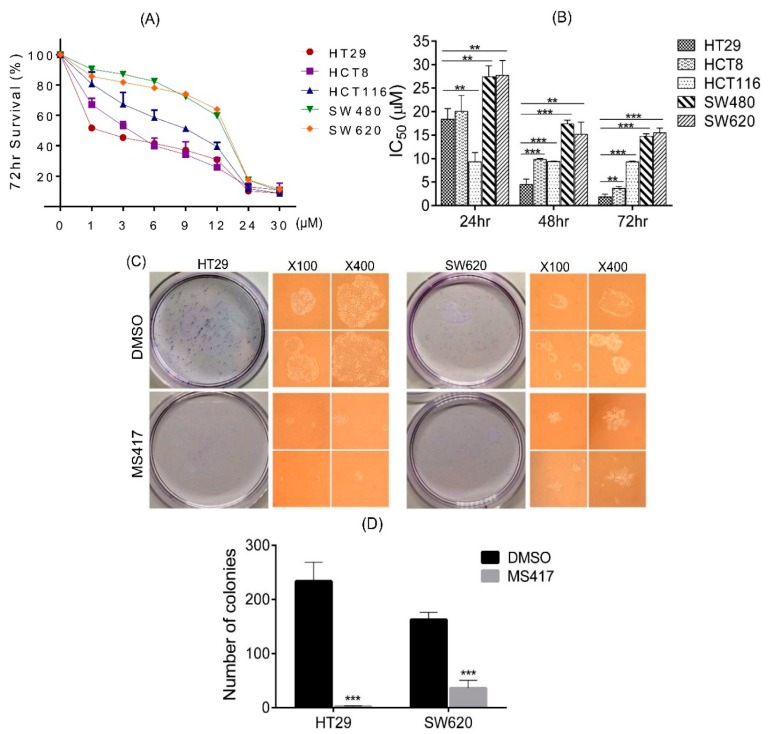

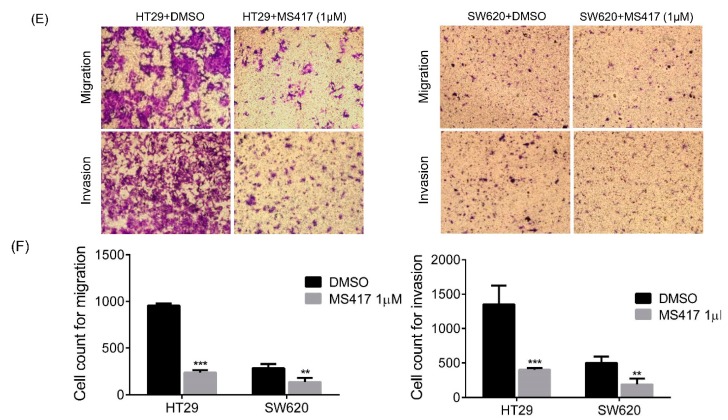
BRD4 inhibition attenuates proliferation, migration and invasion* in vitro*. (**A**) 72 h survival rate curve of HT29, HCT8, HCT116, SW480 and SW620 treated with vehicle (DMSO) or MS417 (1, 3, 6, 9, 12, 24, 30 µM), measured by MTT; (**B**) IC_50_ of HT29, HCT8, HCT116, SW480 and SW620 following vehicle/MS417 for 24, 48 and 72 h; (**C**,**D**) macroscopic and microscopic images and quantification of colonies formed by HT29 or SW620 cell lines treated with MS417 (1 µM); (**E**,**F**) Cell migration and invasion of HT29 or SW620 cell lines were inhibited by MS417 (1 µM). *n* = 3 repeats with similar results. ** *p* < 0.01; *** *p* < 0.001. Values are depicted as Mean ± SEM.

Several different small molecules have been synthesized in recent years with the capability of selectively binding to the bromodomains of BRD4, inhibiting its function [15,16,17]. MS417 is one of them, and has been demonstrated to be effective in other reports [23,27,28,29]. After having observed that BRD4 is highly expressed in CRC, we next sought to determine the consequences that would follow from its inhibition. For this purpose, we treated five distinct colon cancer cell lines (HT29, HCT8, HCT116, SW480 and SW620) with a series of doses of MS417 ranging from 1 to 30 μM, and then plotted out their survival rates relative to the concentration as determined by MTT (3-(4,5-dimethylthiazol-2-yl)-2,5-diphenyltetrazolium) assay. Interestingly, survival rates varied dramatically between the various cell lines at low concentrations, with the survival rate of SW620 cells being double that of HT29 cells at a MS417 concentration of 1 μM (Figure 2A). Overall, HT29 showed the highest sensitivity to MS417 at all doses and all time points tested, whereas SW620 showed the lowest sensitivity in responding to BRD4 inhibition. All cell lines tested responded in a dose dependent fashion, with all of them experiencing near 100% inhibition at a concentration of 12 μM (Figure 2A). Except in HCT116 cell line, for which the population remained consistently from 24 to 72 h, all cell lines also showed time dependent reduction at 24, 48 and 72 h incubations, as expressed as doses(Figure 2B). However, there appeared to be no obvious correlation between the amount of BRD4 protein expressed in a cell line and the effect of the MS417. Because of the striking differences in survival rates over the experimental MS417 treatment concentration range (1–12 μM) despite similar characteristics in several respects (such as with significant expression of the Myc oncogene, an enhancing element found to interact strongly with BRD4) (Table S1), HT29 and SW620 were chosen for further investigation.

To confirm those results, we performed colony formation assay to further clarify the anti-proliferative effects of MS417. As expected, the number of colonies of HT29 and SW620 decreased sharply in the presence of 1 μM MS417 (Figure 2C,D). The colonies that did develop in the MS417 treated groups were also smaller (Figure 2C). In fact, the MS417-treated HT29 group developed almost no colony growth, exhibiting a stronger response to MS417 than SW620 cells, results consistent with our MTT. Overall, our data also suggest that inhibition of BRD4 has potent antiproliferative effects on colon cancer cells, however with an unknown correlation of different cell types.

### 2.3. Colon Cancer Cell Migration and Invasion Are Reduced by BRD4 Inhibition in Vitro

Both HT29 and SW620 are invasive cancer cells with significant metastatic potential [30,31]. Consequently, we performed migration and invasion assay using transwell to verify if MS417 also attenuates the metastatic capability of these lines. After treatment with MS417 for 48 h, cell counts for both HT29 and SW620 reduced significantly compared to control, indicating a significant reduction in cellular movement. The migratory and invasive behavior of HT29 cells was largely curtailed as a result of the addition of MS417. Although less noticeable change was discovered in the SW620 cell line, the reduction of migration and invasion were still statistically significant (Figure 2E,F). While the cell counts may have also been influenced by the toxic effect of MS417, there was no evidence that the cellular debris was blocking migration, and the magnitude of the difference in counts between treated and untreated groups makes the results statistically significant regardless. Based on data above, BRD4 inhibition via MS417 appears to capably suppress CRC cell migration and invasion, suggesting that BRD4 plays a key role in these processes.

### 2.4. BRD4 Inhibition Alters Protein Expression in CRC Cells

**Figure 3:**
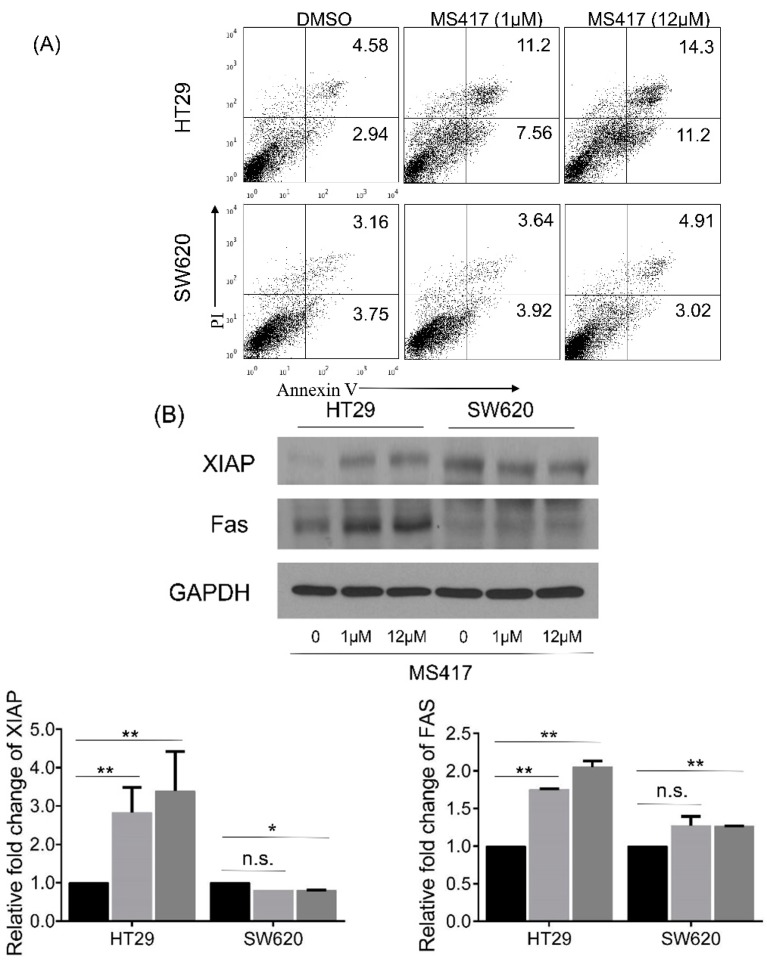

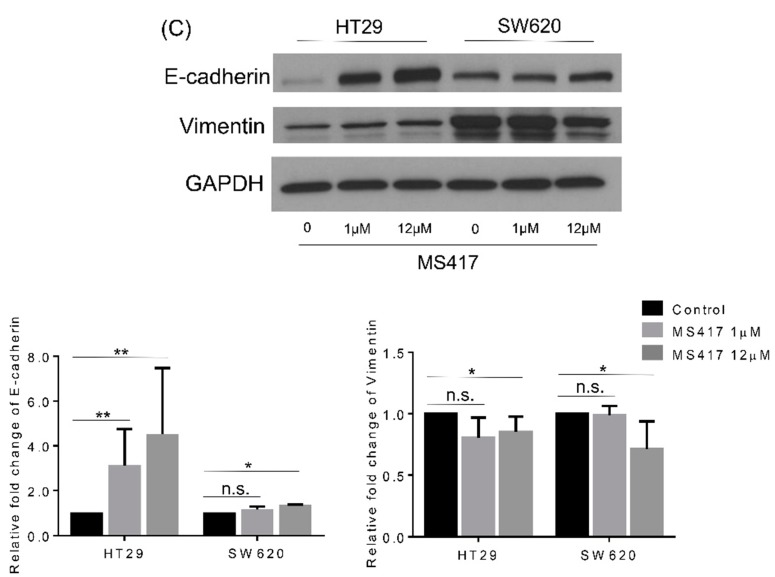
BRD4 inhibition impacts transcriptional programs that control cell proliferation and EMT. (**A**) Apoptosis of HT29 and SW620 cell lines treated with vehicle (DMSO), MS417 (1 µM) or MS417 (12 µM); (**B**) Protein levels of candidate apoptosis-related factors following vehicle/MS417 treatment (1 and 12 µM) for 48 h; (**C**) Protein levels of E-cadherin and Vimentin in HT29 or SW620 cell lines treated with vehicle or MS417 (1 and 12 µM). The expression level of each protein was normalized against GAPDH. * *p* < 0.01; ** *p* < 0.001; n.s. not significant.

Having seen that BRD4 inhibition leads to the reduction of the proliferative ability of CRC cell lines HT29 and SW620, we next tried to elucidate the means by which that effect was generated. We first investigated whether or not the cells were experiencing increased apoptosis as a result of MS417 application. Flow cytometry staining with standard apoptosis markers Annexin V and PI showed minimal increases in the number of apoptotic cells (Figure 3A). Mixed results were observed through Western blotting, with Fas, an important cell surface receptor protein of the TNF receptor family, which induces apoptosis on binding Fas ligand, increased after BRD4 inhibition in both cells (Figure 3B). Interestingly, increased expression of some anti-apoptotic factors (such as XIAP) was also observed in HT29. However, the expression of XIAP decreased in SW620 (Figure 3B). It seems clear that apoptosis was not the controlling factor for BRD4-inhibition mediated suppression of the cells. This result is not surprising, given that cancer cells are generally not believed to be cleared via apoptosis.

We then searched for alternative changes that could explain the dramatic reduction of migration and invasion has been seen in HT29 and SW620 after BRD4 inhibitor treatment. As mentioned previously, we had noticed a curious change in the shape and size of the cells as a result of BRD4 inhibition from the colony growth assays. Based on those results, we investigated for changes in structural proteins, and in proteins involved in epithelial-mesenchymal transition (EMT). Results revealed that protein level of epithelial marker E-cadherin increased sharply in HT29 even at a low dose as 1 μM, while it only showed a significant increase at a higher dose in SW620. These results were consistent with our findings in previous drug sensitive tests, which HT29 present the highest sensitivity to MS417 (Figure 3C). Expression level of vimentin, a mesenchymal marker, which is required for EMT, only decreased at a high dose in both cell lines (Figure 3C). These results confirmed our theory that the inhibition effect of BRD4 inhibitor on cell migration and invasion is at least in part due to EMT inhibition.

### 2.5. BRD4 Inhibition Impairs Colon Cancer Tumor Growth in Vivo

**Figure 4:**
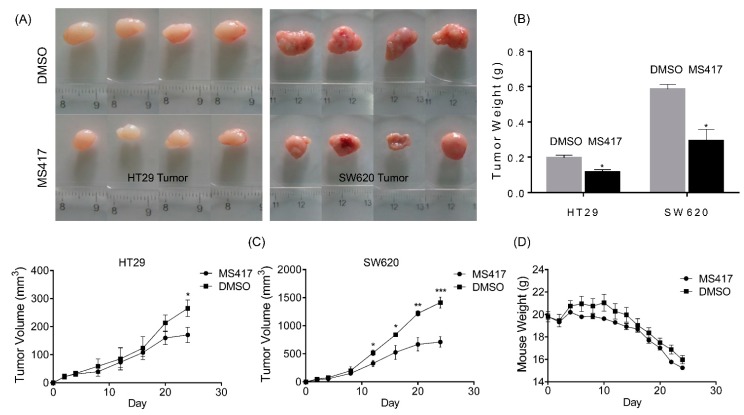
BRD4 inhibition impairs CRC tumor growth* in vivo*. (**A**) Macroscopic images of resected tumors at the conclusion of the experiment; (**B**) average weight of resected tumors; (**C**) average tumor volume of mice injected with either vehicle (DMSO) or MS417 (20 mg/kg, *n* = 4/treatment); (**D**) average mice weights with every 2-day injection of vehicle/MS417 over a time course of 3 weeks. *, *p* < 0.05; **, *p* < 0.05; ***, *p* < 0.001. Values are depicted as Mean ± SEM.

To further examine the antitumorigenic potential of BRD4 inhibition* in vivo*, we next used a classical xenograft mouse model. Nude mice were grafted via injection of HT29 or SW620 into the left or right flanks. Mice then received treatment (MS417 at 20 mg/kg) or sham (DMSO) over the course of three weeks, with the first treatment beginning on the second day. By tracking the development of tumor volume and tumor weight, we found that mice treated with BRD4 inhibitor displayed a significant reduction in tumor growths of both cell lines (Figure 4A,B). Interestingly, tumors in SW620 cells were also largely reduced by MS417, indicating that SW620 cells also showed a high sensitivity to BRD4 inhibitor* in vivo* (Figure 4C), in contrast with a relatively much lower sensitivity in MTT assay. The high degree of proliferation of SW620s in the group treated with sham further demonstrates that the lineage behaves very differently* in vivo* as compared to* in vitro*. HT29 cells, on the other hand, displayed similar growth and sensitivity both* in vivo* and* in vitro*, and HT29 tumors in the treated mice were also highly suppressed (Figure 4C). Overall health effects on the mice were also monitored over the course of MS417 treatment, with mice weights taken at every treatment time. Our results show that group of mice treated with MS417 also had more stable weights as compared with the placebo group (Figure 4D). The changes were pronounced for both injected cell lines. Collectively, these results indicate that MS417 may effectively clear tumor cells* in vivo* without fatal consequences.

### 2.6. BRD4 Inhibition Suppressed Colon Cancer Liver Metastasis in Nude Mice

**Figure 5:**
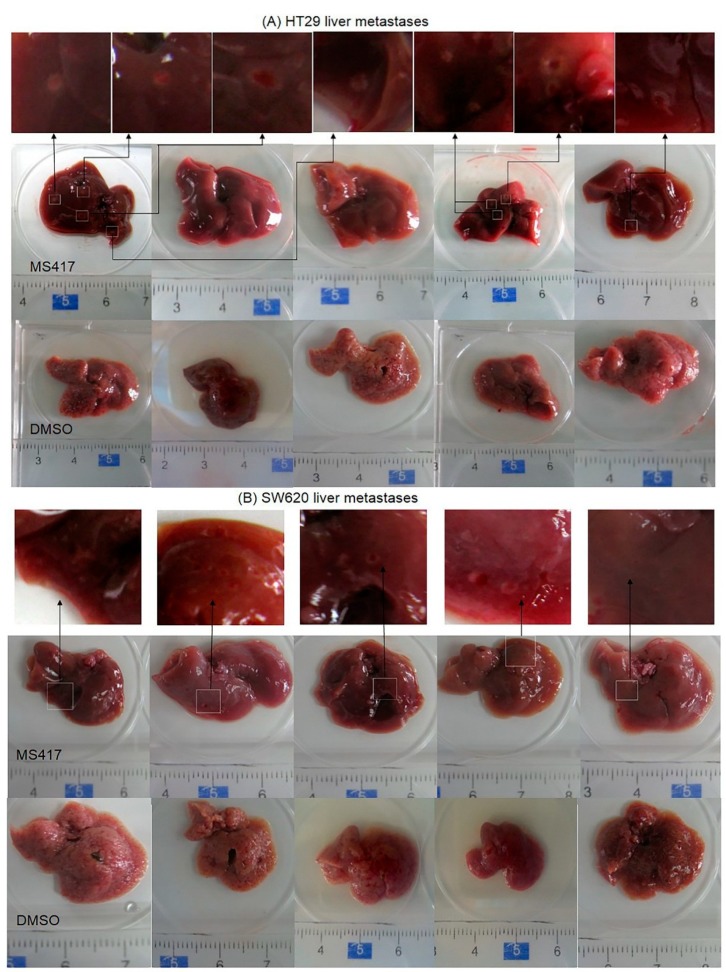

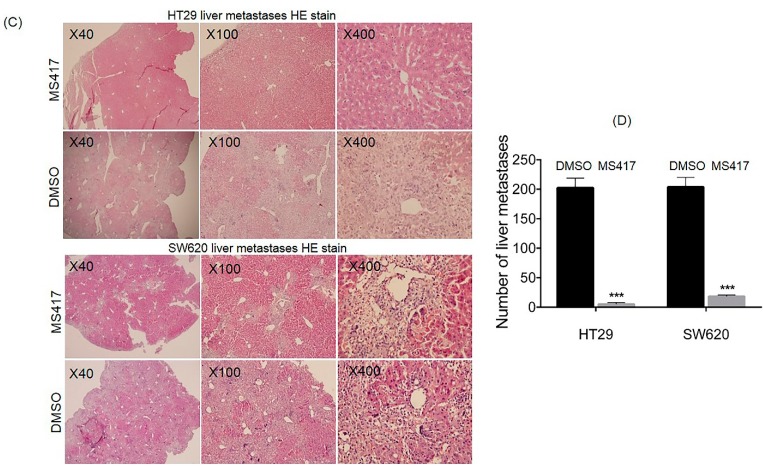
BRD4 inhibition suppresses CRC liver metastasis* in vivo*. (**A**,**B**) Macroscopic images of resected livers at the conclusion of the experiment in mice injected by HT29 or SW620 cell lines and then treated by vehicle/MS417 (20 mg/kg, *n* = 5/treatment); (**C**,**D**) Representative microscopic H&E images of livers. Metastatic focus is circled. *** *p* < 0.001. Values are depicted as Mean ± SEM.

Liver metastasis commonly occurs in conjunction with CRC, and is obviously highly detrimental. It was also a natural extension of our investigation, given that metastasis requires EMT and intracellular communication. Based on the promising effect BRD4 inhibition had on curtailing the migration and invasion of colon cancer cell lines* in vitro* and our results indicating changes it induced in EMT, we next used a liver metastasis model to further confirm our results* in vivo* using HT29 and SW620 cells. BRD4 inhibitor MS417 was administered by intraperitoneal injection every two days, starting two days after tumor cell inoculation into the spleen. After three weeks, all of the mice injected with SW620 developed liver tumors. In the HT29 group however, only 40% of the mice treated with MS417 exhibited liver metastasis, while all of the ones treated with sham (DMSO) developed tumors (Figure 5A,B). Both cell lines showed significant inhibition of metastatic capability with the number of metastases in liver dropping by over an order of magnitude, and fewer liver micrometastases per liver section, following BRD4 inhibition (Figure 5C,D). This result was somewhat unsurprising, since tumor metastasis relies on significant intercellular communication and signal propagation. The complete blockage of metastasis of HT29 in some mice treated with BRD4 inhibitor clarifies the need for BRD4 in EMT. Collectively, these results further support our hypothesis that the* in vitro* effect of EMT and signaling suppression following BRD4 inhibition is also present* in vivo*.

## 3. Discussion

The development and optimization of small-molecule modulators of epigenetic targets as cancer therapeutics is a major collaborative focus of current biomedical research groups. BRD4, an important transcription mediator, binds to acetylated histones to influence transcription and thus stimulate or inhibit gene expression [11,32]. BRD4 is implicated in transmitting epigenetic memory and functions as an associated factor of the positive transcriptional elongation factor b (P-TEFb), which plays an important role in cell cycle progression [33,34]. In fact, BRD4 is expressed ubiquitously in different tissues and is indispensable in cellular growth. Knockout of Brd4 in mice is embryonic lethal, and mouse BRD4 knockout embryonic stem cells do not growth in culture [11,35].

In most previous studies, BRD4 is thought to exist in two spliced isoforms with opposing roles in tumor growth and progression [20,21,22]. They provided the evidences that BRD4 long isoform reduced metastasis, however short isoform enhanced metastasis in breast cancer through interactions with the metastasis susceptibility protein RRP1B and SIPA1 [22]. They further demonstrated that BRD4 short isoform is a nuclear membrane-associated protein, while the long isoform is associated with the nuclear matrix and the short isoform might be an indirect competitive inhibitor of the long isoform [22]. Thus, we tested mRNA expression level of both BRD4 short and long isoforms in CRC. Unexpectedly, both isoforms showed a higher expression in various colon cancer cell lines comparing to normal colon epithelial cell. On the other hand, we observed a higher protein expression level of BRD4 in 45 colon cancer tissues, which were collected from China. It is interesting that the molecule weight of BRD4 antibody used in this study is around 150 kDa, while the full length of BRD4 is supposed to encode a 1362 amino acid protein with a molecular weight of 200 kDa [9,11]. If so, further structure analysis of BRD4 isoforms would be necessary for revealing the association between BRD4 expression level and its role in colon cancer tumorigenesis and metastasis, although we have found that BRD4, most likely the short isoform, has an increasing expression in both colon cancer cell lines and colon cancer tissues, indicating a pro-oncogenic role in CRC.

Given the rationale evidence above, we next hypothesized that CRC might be inhibited by BRD4 inhibitor. Not surprisingly, reduction of cell proliferation by MS417, a novel BRD4 inhibitor, provides a clear indication that BRD4 may be closely related to CRC tumorigenesis. A restrained migratory and invasive capability of colon cancer cells inhibited by MS417 reveals that BRD4 plays a critical role in metastasis, although this does not mean that BRD4 is the only protein that functions importantly in this progression. During the past decades, small-molecular inhibitors of BRD4 are becoming the subject of intensive investigation. MS417 was first designed to block BRD4 binding to the acetylated NF-κB, which effectively attenuates NF-κB transcriptional activation of pro-inflammatory genes [27]. MS417 is structurally related to I-BET and shares the same thieno-tria-zolo-1,4-diazepine scaffold as JQ1. The main difference between MS417 and JQ1 is that the former consists of a methyl ester, which is much smaller than the replacement in latter so that it can interact with Leu-95, Tyr-97 and Tyr-139 in a small hydrophobic cavity formed between the ZA and BC loops [27]. MS417 has been reported to have efficacy effect on HIV-associated kidney disease and recently has been indicated in sustaining melanoma proliferation [23,27,28,29]. We have paid close attention to several previous studies, which showed that BRD4 inhibitor seemed to be effective only at inhibiting primary tumor growth. I-BET151 has been showed unable to reduce pulmonary metastasis in breast cancer and the decrease of lung metastasis burden of melanoma by MS417 has the similar result [22,23]. However, in the present paper, we observed a sharp inhibition of invasiveness and migration properties in colon cancer cell lines, as well as a significant reduction of metastasis* in vivo*. Our results suggest several plausible explanations. First, different isoforms of BRD4 may exist in different cancers and the account of each isoform may vary in different cell lines, which is consistent with our study that both short isoform and long isoform express in colon cancer cells; Second, we couldn’t make a conclusion that the two isoforms have opposing functions, but it is possible that they might somehow be controlled and regulated by each other. Overexpression and reduction of any isoform will result in disorder and disfunction in both; Third, the two isoforms might be not inhibited equally by the inhibitor. Since previous study has demonstrated that BRD4 short isoform has an expanded histone binding range compared to long isoform. It is also reported that casein kinase II phosphorylation may induce conformational changed in BRD4 long isoform that alters bromodomain II accessibility, which may result in a differential activity to BRD4 inhibitors. It is no doubt that further investigation will be required to determine the mechanism in interaction between BRD4 isoforms and inhibitors.

Colorectal cancer, like many other adenocarcinomas, often spreads throughout the body via EMT, and the discovery of distant metastasis has been strongly correlated with worsened prognosis—current AJCC staging classifications of CRC deem a positive test for metastasis to be indicative of progression to stage IV. As a result, interventions that can control tumor spread and limit EMT are of great utility in treating CRC. As other results as well as ours have shown, suppression of these communication channels can strongly inhibit the ability of tumors to metastasis, a result that is of great benefit if achieved in clinical practice [36].

It is possible, of course, that such EMT and signaling disruption was caused in part by the necrosis induced by BRD4 inhibition. However, our result suggest that necrosis alone could not fully account for the disruption, as the disruption could be seen on a protein level even at fairly low concentrations of MS417, when the survival rate of the cells was near full. These protein level changes were reflected in both* in vitro* and* in vivo* results. Future studies may uncover more precisely the exact contributions of the inhibition of BRD4 and necrosis to the results observed. BRD4 is a multifunctional protein, and its potential interaction with proteins involved in EMT and intercellular signaling are not completely unexpected. Work done by other groups has demonstrated that Twist, an EMT mediator, interacts with BRD4 after deacetylation [37]. Other groups have shown that the *C*-terminus domain is critical for BRD4 induction of EMT [21]. These results may be of use in conceiving new therapies for CRC that might use BRD4 inhibition to control tumor expansion, making it easier for the tumor to be eliminated in place (likely in conjunction with other chemotherapy medicines). Furthermore, the differences between mice that allow for some to have liver metastasis completely blocked off following BRD4 inhibition merits additional study, as such work may identify novel factors that act in conjunction with BRD4 inhibition to produce that result. Further work will be needed to clarify the exact contributions of each interaction, which BRD4 inhibition may be disrupting in CRC. After all, other proteins besides Twist, such as Snail and Zeb1, have been found to work as transcription activators to up-regulate EMT [38,39], and may also be interaction partners with BRD4.

Given the increasing popularity of multi-drug chemotherapy regimens, the identification of additional functional roles and pathways provides essential information for therapy design. The anti-metastatic effect of BRD4 may allow for it to fill a valuable function in anti-CRC treatments. For instance, the anti-foliate medication methotrexate is in widespread use for its tumorcidal abilities [40,41]. However, folate deprivation has also been demonstrated to promote CRC cell invasion [42]. A BRD4 inhibitor may potentially serve to suppress induced invasion, as an alternative to simply increasing the dosage of methotrexate. In addition, several commonly used chemotherapy drugs, such as paclitaxel have been demonstrated to also induce resistance, through the promotion of inflammation along the NF-κB pathway [43]. BRD4 inhibition has been shown to capably suppress NF-κB transcription and function [17,27]. Given the constant search for effective therapies that are effective at lower doses of medication (in the interest of limiting side effects), such potential combinations may have useful clinical applications.

In summary, our findings demonstrate that BRD4 is a positive regulator in colorectal cancer, playing a key role in tumor metastasis. We demonstrate that one BRD4 inhibitor is effective in suppressing cancer growth and preventing distal metastasis through multiple means. The latter role may allow BRD4 inhibitors to be suitable additions to existing chemotherapy regimens in colorectal cancer.

## 4. Experimental Section

### 4.1. Cell Culture

All cell lines were purchased from the American Type Culture Collection (ATCC, Manassas, VA, USA). The human normal colon epithelial cells (FHC cell line) were grown in a DMEM:F12 Medium (ATCC) supplemented with 10% heat-inactivated bovine fetal serum (FBS; Gibco, Grand Island, NY, USA), 100 U/mL penicillin, and 100 µg/mL streptomycin (Invitrigen, Camarillo, CA, USA), extra 10 mM HEPES (Gibco), 10 ng/mL cholera toxin (Sigma-Aldrich, St. Louis, MO, USA), 0.005 mg/mL transferrin (Sigma-Aldrich) and 100 ng/mL hydrocortisone (Sigma-Aldrich). The human colon cancer cell lines HT29, LoVo, SW48, SW480, HCT8, HCT116 and SW620 were maintained in Dulbecco’s modified Eagle’s Medium (DMEM) with 10% FBS, 100 U/mL penicillin and 100 µg/mL streptomycin. Cells were cultured at 37 °C in an incubator containing 5% CO_2_.

### 4.2. Patient Samples

This study included 45 paired colorectal cancer samples (Table 1), which were obtained from patients who underwent colonoscopy between January 2011 to January 2012 at Southern Medical University, Guangzhou, China. This study was reviewed and approved by the Institution Ethical Review Boards of Southern Medical University. Samples were kept at −80 °C until analysis. The histological diagnosis of resected specimens was confirmed by at least two well-trained pathologists. Total protein from each sample was extracted as following: 1 mL of 1× lysis buffer (Cell Signaling Technology, Danvers, MA, USA) with 1× protease inhibitor cocktail (Roche Applied Science, Indianapolis, IN, USA) and 1× phosphatase inhibitor cocktail was added to each tissue sample and the lysate was sonicated twice for 15 s each time on ice, and then centrifuged at 14,000 rpm for 30 min at 4 °C. The protein concentration was determined using BCA Protein Assay kit (PIERCE, Rockford, IL, USA) and analyzed via Western blotting.

### 4.3. Real-Time PCR

Total RNA was extracted using TRIzol reagents (Life Technologies, Carlsbad, CA, USA), according to the manufacturer’s instructions. RNA samples were reverse transcribed at 50 °C for 50 min, using the high-capacity cDNA reverse transcription kit (SuperScript; Life Technologies, Carlsbad, CA, USA) according to the standard protocol of the supplier. Sequences of the PCR primers used are as follows: β-actin primer forward: 5'-CCAGAGCAAGAGAGGCATCCT-3' and reverse: 5'-TAGATGGGCACAGTGTGGGTGA-3'; BRD2 primer forward: 5'-GTCAAACTGG GTCTACCGGATT-3' and reverse: 5'-CTTTTCCAGCGTTTGTGCCA-3'; BRD4 short isoform primer forward: 5'-ACAACAAGCCTGGAGATGACA-3' and reverse: 5'-GTTTGGTACCGTGGAAACGC-3'; BRD4 long isoform primer forward: 5'-CCGGAAATGAAGCCTGTGGAT-3' and reverse: 5'-TTTTCA GGTCCTTTTTGGGCG-3'; Commercial BRD4 primer (Qiagen, Valencia, CA, USA) was used as positive control. Real-time PCR was carried out using LightCycler^®^ 480 Real-Time PCR System (Roche Diagnostics, Penzberg, Germany). Amplification system included 15 μL of SYBRGreen Mix (10 μL), ddH2O (1 μL), cDNA (2 μL), forward primer (1 μL) and reverse primer (1 μL). The results of real-time PCR were analyzed by the DCT method: Δ*C*t = *C*_t__selected gene_ − *C*_t__β-actin_, RQ (Relative Quantitation) = 2^−Δ*C*t^ × 100%. The results of real-time PCR were presented as the ratio between the selected genes and β-actin transcripts. All experiments were performed in triplicates.

### 4.4. MTT Assay

MTT assay was performed to assess the IC_50_ of BRD4 inhibitor. Cells were seeded at 2 × 10^3^ cells per well on a 96-well plate. The day after (day 0), cells were treated with DMSO or with increasing doses of MS417 (1, 3, 6, 9, 12, 24, 30 µM) and cultured for another 3 days. The 3-(4,5-dimethylthiazol-2-yl)-2,5-diphenyltetrazolium (MTT) solution as added to each well and then the plates were cultured for 2 h at 37 °C. The Medium was removed and cells were stained with 0.1% crystal violet. Crystals were dissolved in DMSO and optical density was read at 590 nm. Experiments were repeated three times, and data represented as the mean of sextuplicate wells ± SEM.

### 4.5. Colony Formation Assay

Cells were seeded at 250 cells per well in 6-cm well plates (*n* = 3). Cells were then treated with MS417 after 1 day, and culture cells over 10 days. The cells were then stained with crystal violet, photographed, and counted.

### 4.6. In Vitro Migration and Invasion Assay

Equal numbers of cells (3 × 10^5^) in DMEM complemented with 2% FBS were added to the upper compartment of a Transwell chamber (8 µm, Costar, Cambridge, MA, USA) and kept at 37 °C for 48 h. As a chemoattractant, the lower compartment contained DMEM supplemented with 10% FBS. At the end of the incubation period, cells from the upper surface of the filter were wiped off with a cotton swab. Cells on the lower surface of the filter were fixed with 2% formaldehyde for 30 min and then stained with 0.1% crystal violet. The number of cells having migrated to the bottom of the chamber was counted in the light microscope on ten randomly selected fields. The mean number of cells was calculated per field. Three sets of experiments were carried out, each in triplicate. For the matrigel assay, the filter was replaced by a BD BioCoat Matrigel invasion chamber (BD Bioscience, Mountain View, CA, USA).

### 4.7. Annexin V/PI Assays for Apoptosis

For Annexin V/PI assays, cells were stained with Annexin V-FITC and PI, and evaluated for apoptosis by flow cytometry according to the manufacturer’s protocol (BD PharMingen, San Diego, CA, USA). Briefly, 1 × 10^6^ cells were washed twice with phosphate-buffered saline (PBS), and stained with 5 µL of Annexin V–FITC and 10 µL of PI (5 µg/mL) in 1× binding buffer (10 mm HEPES, pH 7.4, 140 mm NaOH, 2.5 mm CaCl_2_) for 15 min at room temperature in the dark. The apoptotic cells were determined using a flow cytometer (FACSCalibur, BD Biosciences). Both early apoptotic (annexin V-positive, PI-negative) and late (annexin V-positive and PI-positive) apoptotic cells were included in cell death determinations.

### 4.8. Western Blot Analysis

HT29 and SW620 cells were seeded onto 10 cm dish at a density of 1 × 10^6^. The day after, cells were completely adherent and then treated with MS417 at doses of 1 and 12 µM or with DMSO as control. Keep culturing for 48 h, cells were harvested by the end of the experiment. Proteins were extracted as descrbed as before. For Western blot assay, proteins (20 µg) were boiled for 5 min and resolved by 10% SDS-polyacrylamide gel electrophoresis (SDS-PAGE). Proteins were then transferred from gels to nitrocellulose membranes. The membranes were blocked with 3% BSA for 1 h at room temperature and incubated with primary antibodies at 4 °C overnight. All primary antibodies used were purchased from Santa Cruz Biotechnology (Santa Cruz, CA, USA). The second day, membrane was incubated with secondary antibodies conjugated with horseradish peroxidase (Amersham, Arlington, Height, IL, USA) for 1 h at room temperature. The membrane was developed with chemiluminescence substrate (Immun-Star™ HRP Peroxide Buffer/Immun-Star™HRP Luminol Enhancer) (Bio-Rad, Hercules, CA, USA). The grey value of each protein was measured using freeware ImageJ and normalized by the standard control protein glyceraldehyde-3-phosphate dehydrogenase (GAPDH).

### 4.9. Mouse Xenograft and Liver Metastasis Model

All procedures involving animals were approved by Animal Care and Use Committee (Approval number SYXK 2011-0074) of Southern Medical University. Surgery was performed using sodium pentobarbital anesthesia. Twenty-eight female BALB/c-nu nude mice (16–20 g) aged 5–7 weeks were obtained from Guangdong Province Medical Experimental Animal Center and were used to build two models. HT29 cells and SW620 cells were injected (5 × 10^6^/Mouse) subcutaneously in the left and right flanks of nude mice. Two days after, mice were randomized into 2 groups, 4 mice per each group and treated intraperitoneally with vehicle (2.5% DMSO) or with 20 mg/kg MS417 every 2 days. Tumor volume and mouse weight were measure every 2 days during 3 weeks. Tumor weight was analyzed when the tumors were excised at the end of the experiment. For the liver metastasis model, HT29 cells or SW620 cells were transplanted (1.5 × 10^6^) intrasplenically. MS417 or DMSO were administered by intraperitoneal injection every 2 days, starting 2 days after tumor cell inoculation. After treating the mice with MS417 or placebo for 3 weeks, liver was removed and metastatic tumors on surface of liver were measured. Afterward, tissues were fixed in 10% formalin, paraffin embedded, and 5 µm sections were hematoxylin and eosin (H&E)-stained.

### 4.10. Statistical Analysis

Statistical analyses were performed with MS Excel and GraphPad Prism (5.01 for Windows; GraphPad Prism Software Inc., San Diego, CA, USA), version 17.0 (SPSS, Inc., Chicago, IL, USA). Mean values ± SEM are representative of one of three independent experiments. Statistical significance was determined by unpaired *t* test. Of note, * *p* < 0.05; ** *p* < 0.01 and *** *p* < 0.001.

## 5. Conclusions

A multifunctional protein that can influence the behavior of many proteins, BRD4 has been the subject of significant scrutiny. In our present paper, we suggest that BRD4 has a role in colorectal cancer and epithelial-mesenchymal transition, in addition to the previous functions discovered. We also report the success of using BRD4 inhibitor to target colorectal cancer cell growth *in vivo*, albeit through xenografts. Further confirmation of these phenomena may help to further inform future use of BRD4 inhibitors, and guide the testing of additional compounds for use in specialized chemotherapy regimens. Future studies may also identify other proteins of interest related to these proteins altered during the course of BRD4 inhibition.

## Supplementary Materials

Supplementary materials can be found at http://www.mdpi.com/1422-0067/16/01/1928/s1.
